# *PYCARD* gene polymorphisms and susceptibility to periodontal and coronary heart diseases

**DOI:** 10.25122/jml-2023-0263

**Published:** 2024-02

**Authors:** Zina Ali Daily, Batool Hassan Al-Ghurabi, Ahmed Makki Al-Qarakhli

**Affiliations:** 1 Department of Periodontics, College of Dentistry, University of Baghdad, Baghdad, Iraq; 2 Department of Periodontics, College of Dentistry, University of Al-Ameed, Karbala, Iraq; 3 Department of Basic Science, College of Dentistry, University of Baghdad, Baghdad, Iraq; 4 Department of Oral Diagnosis, College of Dentistry, University of Anbar, Ramadi, Iraq

**Keywords:** periodontitis, coronary heart disease, genetic variation, *PYCARD* genes, SNPs, Single Nucleotide Polymorphisms, CHD, Coronary Heart Disease, MI, Myocardial Infarction, PLI, Plaque Index, PCR, Polymerase Chain Reaction

## Abstract

Numerous studies have established a link between gene variants within the inflammasome complex and the incidence of periodontitis and cardiovascular illness across various ethnic groups. This study investigated the association between *PYCARD* gene polymorphism and susceptibility to periodontal disease and coronary heart disease (CHD) and their correlation with clinical periodontal indices. A total of 120 participants were enrolled, categorized into four groups: 30 healthy controls (C), 30 patients with generalized periodontitis (P), 30 patients with atherosclerotic CHD but clinically healthy periodontium (AS-C), and 30 patients with both atherosclerotic CHD and generalized periodontitis (AS-P). We recorded demographic data, collected blood samples, and measured periodontal indices, including plaque index, clinical attachment loss, bleeding on probing, and pocket depth. The genomic variant of the *PYCARD* gene was analyzed using a conventional polymerase reaction. A significant prevalence of T and G allele mutations and a higher distribution of CT and TT genotypes in *PYCARD* C/T (rs8056505) and the AG genotype in *PYCARD* A/G (rs372507365) were observed in groups P, AS-P, and AS-C. These single nucleotide polymorphisms (SNPs) were also positively correlated with the severity of clinical periodontitis indices. Our findings suggest that the increased frequency of T and G alleles and the distribution of CT, TT, and AG genotypes in *PYCARD* SNPs are significantly associated with an elevated risk for periodontal disease and CHD. These SNPs may participate in the pathogenesis of these conditions. The study reinforces the potential role of these genetic markers as risk factors for both diseases in the Iraqi population.

## INTRODUCTION

Periodontitis is characterized by the progressive degradation of periodontal tissue associated with the buildup of certain oral microorganisms in the dental biofilm, triggering inflammation and abnormal responses within the body. Genetics, age, and lifestyle factors influence the susceptibility to this multifactorial disease, each contributing to the risk profile [1,2]. Coronary heart disease (CHD), which can precipitate myocardial infarction, results from cardiovascular dysfunction, predominantly due to atherosclerosis. This condition is exacerbated by excessive cell death, apoptosis, and inflammation, leading to a necrotic core within atherosclerotic plaques [3]. The *PYCARD* gene encodes an adaptor protein essential for the recruitment of caspase enzymes. The inflammasome complex, comprising proteins and *PYCARD*, initiates a significant inflammatory response by interacting with caspase-1, -8, and -9, potentially leading to apoptosis [4-6]. The inflammasome is activated when membrane pores, created by cleaved Gasdermin fragments, trigger pyroptosis, which subsequently results in the release of inflammatory mediators. Pyroptosis serves an essential role in eliminating pathobionts, facilitating the extracellular release of pathogens during neutrophil-induced cell death. However, it also plays a part in the pathogenesis of various diseases by inducing severe inflammatory responses [7,8]. Genetic polymorphisms within inflammasome genes have been extensively studied [9]. However, few reports have identified a link between *PYCARD* gene variations and cancer diseases [10,11]. To our knowledge, there has been no investigation into the association of *PYCARD* gene polymorphisms and an increased susceptibility to periodontal disease and/or coronary heart disease. Therefore, the current study aimed to investigate the relationship between *PYCARD* SNPs and the risk of developing periodontal disease and/or CHD and to assess the correlation between *PYCARD* SNPs and clinical periodontal indices.

## MATERIAL AND METHODS

This case-control study was conducted across multiple centers in Karbala between March and December 2022. Inclusion criteria were as follows: patients who (a) consented to participate in the study; (b) retained at least two-thirds of their total dentition, free from decay; (c) were generally healthy, except for a cardiac catheterization-confirmed diagnosis of CHD; and (d) exhibited varying severities and rates of progression of periodontal disease. Exclusion criteria included patients with (a) systemic comorbidities, (b) periodontal therapy within the past 6 months, (c) current smokers, (d) use of anti-inflammatory or immunosuppressive drugs within the last 3 months, and (e) those pregnant during the study period.

The genetic markers of the study groups, specifically the polymorphisms in their *PYCARD* genes, were identified based on the prevalence of periodontitis and CHD observed in the primary outcomes of the pilot study. The sample size was calculated using an odds ratio (OR) analysis on the Epitools website, aiming for an 80% power of detection at an alpha level of 0.05. The study included 120 participants, with 30 healthy controls (C), 30 patients with generalized periodontitis (P), 30 patients with atherosclerotic CHD but clinically healthy periodontium (AS-C), and 30 patients with both atherosclerotic CHD and generalized periodontitis (AS-P). Participants were men and women aged 35 to 65, with a body mass index (BMI) less than 25 kg/m^2^.

Patients with periodontitis were diagnosed following the classification by Tonetti *et al*. [12]. Diagnosis involved identifying individuals with a generalized extent of interdental clinical attachment loss (CAL) at two or more non-adjacent teeth or at least 3 mm of CAL on either the buccal (facial) or lingual/palatal surfaces. Additionally, a probing pocket depth (PPD) greater than 3 mm at two or more teeth was required for classification into periodontitis stages III and IV, grades B and C. These stages indicate an unstable status, characterized by either a PPD of 4 mm or more with bleeding on probing (BOP) or a PPD greater than 5 mm, with or without BOP. [13]. Clinical symptoms such as dyspnea, chest pains, electrocardiogram (ECG) changes, elevated blood lipid profiles, and diagnostic percutaneous coronary intervention revealing atheromas exceeding 70% obstruction were used for CHD diagnosis [14-17]. Demographic data were recorded, and blood samples were collected from all participants. Clinical assessments were performed using a UNC-15 probe to measure periodontal indices, including plaque index (PLI) [18], BOP [19], CAL, and PPD. Blood samples comprised 2 ml of venous blood collected in ethylenediaminetetraacetic acid (EDTA) tubes. The genomic DNA was isolated from the blood samples utilizing a ReliaPrepTM Blood gDNA Miniprep System (Promega) kit following the instructions supplied by the manufacturer. DNA concentration was quantified with a Macrogen QuantiFluor dsDNA system. The lyophilized form was dissolved in nuclease-free water to obtain a working primer solution. The primer was examined to determine its optimum degree of heat for annealing. Next, the same pair of primers was used for amplification in the template of DNA, forward, TGTAAAACGACGGCCAG TGAGATGACATGCGTGATGAG, and reverse CAGGAAACAGCTATGACGGCTCTCACTGG GTTTATTG at annealing temperatures. The PCR products were then subjected to agarose gel electrophoresis and Sanger sequencing using a Macrogen ABI3730XL Automated DNA Sequencer. Intra-examiner reliability was verified through two calibration sessions involving five patients with periodontitis, conducted one hour apart. Kappa coefficient values for BOP and PLI were 0.87 and 0.92, respectively, while intraclass correlation coefficients for PPD and CAL were 0.93 and 0.87, establishing the reliability of the study.

### Statistical analysis

Data analysis was conducted using GraphPad Prism version 8.0 (GraphPad Software Inc). Means and standard deviations for each variable were calculated, and inter-group differences were assessed using one-way ANOVA. Hardy-Weinberg equilibrium was tested for the SNPs in each group, followed by an analysis of allele frequency and genotype distribution using OR. Pearson's correlation coefficient was employed to examine the association between *PYCARD* C/T (rs8056505) and *PYCARD* A/G (rs372507365) gene polymorphisms and periodontal indices.

## RESULTS

### Demographic and clinical findings

The demographic data and clinical characteristics of the study groups are detailed in Table 1. There were no statistically significant differences between the groups in terms of age, gender, and BMI (*P* > 0.05). In the four study groups, the average ages were 55 ± 6.71 for the control group (C), 53 ± 2.4 for the periodontitis group (P), 53.31 ± 3.88 for the CHD with clinically healthy periodontium group (AS-C), and 54 ± 8.4 for the CHD with periodontitis group (AS-P). The male-to-female ratio was 80:20 across groups, with an overall average BMI of 23 kg/m^2^. However, the periodontitis groups (P, AS-P) had higher values for PLI, BOP, PPD, and CAL compared to the control group (C) (Table 1).

**Table 1:** Demographic variables and indices of periodontal in the study groups

Group/ average ± SD	Control (C)	Periodontitis (P)	CHD with clinically healthy periodontium (AS-C)	CHD with periodontitis (AS-P)	Test results	*P* value
Age	55 ± 6.71	53 ± 2.4	53.31 ± 3.88	54 ± 8.4	15.059	0.073
BMI	23.35 ± 0.85	23.17 ± 0.72	23.97 ± 0.31	23.24 ± 0.71	44.209	0.308
Gender Male (*n*, %) Female (*n*, %)	24 (80.0%) 6 (20.0%)	24 (80.0%) 6 (20.0%)	24 (80.0%) 6 (20.0%)	23 (76.7%) 7 (23.3%)	0.152	0.09
PLI	8.34 ± 0.84	53.41±0.31	7.35 ± 1.42	73.42 ± 1.73	403.728	0.02
BOP	4.93 ± 0.37	62.72 ± 0.38	6.82 ± 0.34	67.42 ± 1.84	611.493	0.01
PPD	-	5.12 ± 0.83	-	8.4 ± 0.62	13.534	0.01
CAL	-	7.12 ± 0.74	-	9.12 ± 0.32	10.057	0.04

Statistical analyses were performed using ANOVA for age, BMI, PLI, and BOP, and the independent samples T-test for PPD and CAL. Chi-square test was used for gender distribution. *P* values less than 0.05 were considered statistically significant. PLI, Index of plaque; BOP, Bleeding on probing; PPD, Probing pocket depth; CAL, Clinical attachment loss.

### Polymorphisms in PYCARD gene and periodontal disease and/or coronary heart disease

This study investigated the association between variations (polymorphisms) within the *PYCARD* gene and an increased risk of developing periodontal disease and/or CHD. Two specific polymorphisms were analyzed: *PYCARD* C/T (rs8056505) and *PYCARD* A/G (rs372507365).

### PYCARD C/T (rs8056505)

**Figure 1:**
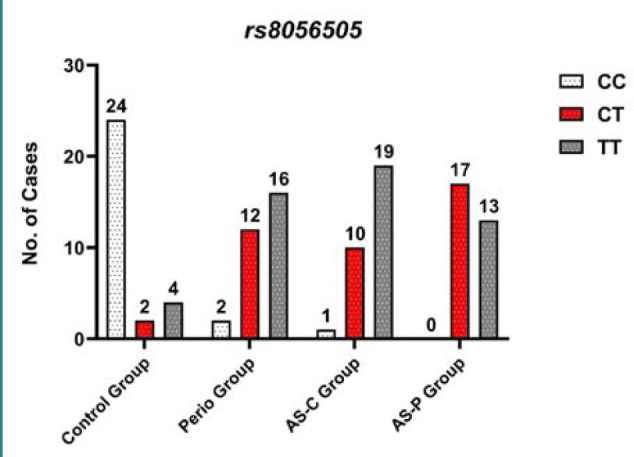
*PYCARD* C/T (rs8056505) genotype distribution among study groups

The *PYCARD* C/T (rs8056505) polymorphism showed a significantly higher frequency of the T allele and the TT genotype in individuals with periodontal disease (P and AS-P groups) compared to the control group (C) group (Table 2, *P* values < 0.05). This finding is supported by the elevated odds ratios (ORs) observed for the TT genotype in the disease groups (Table 2 and Figure 1). For instance, individuals in the periodontitis group with the TT genotype were 13.47 times more likely to have the disease compared to those with the CC genotype (OR, 13.47; 95% CI, 0.04-0.470). This finding aligns with existing research suggesting a higher prevalence of the T allele in Asian populations (0.69) compared to the global average (0.6) based on the Genome Aggregation Database (gnomAD) [20,21].

**Table 2:** Genotypic distribution and odds ratios for *PYCARD* C/T (rs8056505) SNPs across study groups

Genotype	Control	P	*P* value	O.R (95% Cl)	AS-C	*P* value	O.R (95% Cl)	AS-P	*P* value	O.R (95% Cl)
CC	24 80%	2 7%	0.00	56.0 (11.1-260.6)	1 3%	0.00	116.0 14.65-122	0 0%	0.000	229.9 (12.335 -4285.42)
CT	2 7%	12 40%	0.0023	10.72 (0.02-0.470)	10 33%	0.0098	14.29 (0.029- 0.65)	17 57%	0.00	54.33 (0.0117 -0.2757)
TT	4 13%	16 53%	0.001	13.47 (0.04-0.470)	19 64%	0.00	8.91 (0.029-0.31)	13 43%	0.0099	20.12 (0.06497 - 0.720)
C Allele	0.8 50%	0.26 16	<0.000	13.75 (5.51-31.65)	0.2 12%	0.000	20.0 (7.49-47.73)	0.3 17%	0.000	12.65 (5.131 -29.64)
T Allele	0.2 10%	0.74 44%	<0.000	13.75 (5.51-31.65)	0.8 48%	0.000	20.0 (7.49-47.73)	0.7 43%	0.000	12.65 (5.131 -29.64)
HWE X^2^	0.00	0.00			4.646			0.00		
*P* value	>0.9999	>0.9999			0.098			>0.9999		

OR = Odds ratio, HWE X^2^ = Hardy-Weinberg equilibrium

### PYCARD A/G (rs372507365)

**Figure 2:**
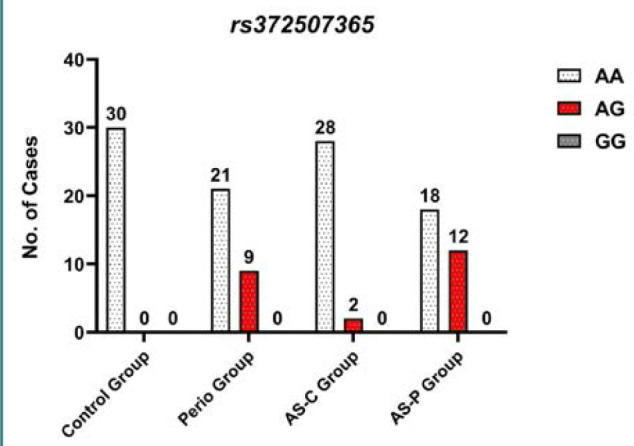
*PYCARD* A/G (rs372507365) genotype distribution among study groups

Furthermore, the *PYCARD* A/G (rs372507365) polymorphism revealed a higher distribution of the AG genotype, with significant odds ratios in the P group (OR = 7.12; 95% CI, 0.0066-0.5678), the AS-C group (OR = 1.869; 95% CI, 0.0086-4.0625), and the AS-P group (OR = 5.83; 95% CI, 0.0454-0.3461), compared to the control group (C). In addition, the GG genotype of the P group had a high OR = 1.0 (0.0192 to 52.0394), with 1.0 (0.0192 to 52.0394) for the AS-C group and 1.0 (0.0192 to 52.0394) for the AS-P group compared to the C group, as illustrated in Table 3 and Figure 2. The polymorphisms of the *PYCARD* A/G (rs372507365) increased the G allele frequency, which was 0.18 in the P group, 0.03 in the AS-C group, and 0.2 in the AS-P group compared to the A allele of the study groups shown in Table 3. The G allele frequency among the Iraqi population is slightly higher than in other Asian countries (0.001) and the global population allele frequency (0.00), according to data from the gnomAD database. This indicates that Asian populations exhibit a broader range of allele frequencies compared to other populations. *PYCARD* C/T (rs8056505) and *PYCARD* A/G (rs372507365) distribution of genotypes had no significant differences in HWE across the study groups (Tables 2 and 3). The association between the *PYCARD* genes and the clinical indices of periodontal disease for the four groups is shown in Table 4.

**Table 3:** Genotypic distribution and odds ratios for PYCARD A/G (rs372507365) SNPs across study groups

Geno-type	Control	Perio	*P* value	O.R 95% Cl	AS-C	*P* value	O.R (95% Cl)	AS-P	*P* value	O.R 95% Cl
AA	30 100%	21 70%	0.004	13.3 (1.761- 149.7)	28 94%	0.2857	5.3509 (0.246-116.31)	18 60%	0.0005	20.0 (2.890-220.2)
AG	0 0%	9 30%	0.004	7.12 (0.006-0.567)	2 6%	0.2857	1.869 (0.008-4.062)	12 40%	0.0005	5.83 (0.0454-0.3461)
GG	0 0%	0 0%	1.00	1.0 (0.019-52.039)	0 0%	1.00	1.0 (0.019- 52.03)	0 0%	1.00	1.0 (0.0192-52.0394)
A Allele	1.0 60%	0.82 51%	0.0018	10.59 (1.596- 117.9)	0.97 58%	0.0268	0.195 (0.041- 0.817)	0.8 48%	0.4711	1.4 ( 0.5560-3.441)
G Allele	0.0 0%	0.18 9%	0.0018	10.59 (1.596- 117.9)	0.03 2%	0.0268	0.195 (0.041- 0.817)	0.2 12%	0.4711	1.4 (0.5560-3.441)
HWE X^2^	0.00	1.61			2.0			1.45		
*P* value	>0.9999	0.4456			0.3679			0.4837		

OR, Odds ratio; HWE X^2^, Hardy-Weinberg equilibrium

**Table 4:** Correlation between *PYCARD* gene variants (rs8056505 C/T, rs372507365 A/G) and periodontal indices in study groups

SNPs		PLI		BOP		PPD		CAL	
	Patients Groups	r Value	*P* value	r Value	*P* value	r Value	*P* value	r Value	*P* value
PYCARD C/T (rs8056505)	P group (*n* = 30)	0.231	0.220	0.447	0.013	0.399	0.029	0.672	0.000
	AS-C group (*n* = 30)	-0.024	0.898	-0.237	0.208	0.00	0.00	0.00	0.00
	AS-P group (*n* = 30)	0.198	0.293	0.498	0.005	0.463	0.030	0.504	0.005
PYCARD A/G (rs372507365)	P group (*n* = 30)	0.093	0.626	0.278	0.137	0.329	0.536	0.364	0.048
	AS-C group (*n* = 30)	0.315	0.090	0.077	0.685	0.00	0.00	0.00	0.00
	AS-P group (*n* = 30)	0.014	0.940	0.128	0.499	0.332	0.073	0.536	0.004

Significant differences (*P* <0.05); r, Pearson correlation coefficient; PLI, Plaque index; BOP, Bleeding on probing; PPD, Probing pocket depth, CAL, Clinical attachment loss.

The association between *PYCARD* genotypes and periodontal clinical indices was assessed using the Pearson correlation coefficient. There was a significant positive correlation between the *PYCARD* C/T (rs8056505) and BOP, PPD, and CAL in the periodontitis group (P) and the CHD group with periodontitis (AS-P), respectively. A significantly positive correlation was also observed between *PYCARD* A/G (rs372507365) and CAL in the periodontitis group (P) and the CHD group with periodontitis (AS-P), respectively.

## DISCUSSION

This study investigated the association between *PYCARD* gene polymorphisms (C/T rs8056505 and A/G rs372507365) and susceptibility to periodontal disease and coronary heart disease in an Iraqi population. The findings suggest that specific genotypes in these polymorphisms might be linked to an increased risk of these diseases. Furthermore, this study established a significant association between *PYCARD* gene polymorphisms and periodontal indices. The data in Table 2 shows that individuals with the TT genotype of the *PYCARD* C/T (rs8056505) polymorphism are significantly more likely to have periodontitis, CHD and CHD with periodontitis compared to those with the CC genotype (OR = 13.47; 95% CI, 0.04-0.470), (OR = 8.91; 95% CI, 0.029-0.31) and (OR = 20.12; 95% CI, 0.06497-0.720) respectively. C allele carriers have a lower chance of contracting both diseases, indicating that the *PYCARD* C/T (rs8056505) SNP is linked to an elevated risk of periodontal disease and atherosclerotic CHD. This suggests that individuals with the TT genotype are over 13 times more likely to have periodontitis, over 8 times more likely to have CHD, and over 20 times more likely to have CHD with periodontitis compared to those with the CC genotype. The AG genotype of the *PYCARD* A/G (rs372507365) polymorphism revealed a statistically significant association with periodontal disease and atherosclerotic CHD with periodontitis susceptibility (OR = 7.12; 95% CI, 0.0066-0.5678) and (OR = 5.83; 95% CI, 0.004540-0.3461) in this study population as compared to the AA genotype (Table 3). A allele carriers had a lower chance of contracting both diseases. This suggests the AG genotype is associated with an increased risk of periodontal disease and atherosclerotic CHD.

In summary, this study investigated *PYCARD* gene variations SNPs located in the promoter region, a crucial area of the 5' untranslated region (UTR) of DNA that contains regulatory elements like the proximal promoter. These regulatory elements influence gene expression in immune cells and tissue-resident cells. The SNPs might disrupt these elements, potentially leading to epigenetic modifier loss and altered expression of *PYCARD* proteins. This, in turn, could be linked to pyroptosis, a form of programmed cell death. Interestingly, similar associations between *PYCARD* variations and altered gene expression have been observed in various cancers, including lung cancer [22], breast cancer [23], schwannoma tumor [4], prostate cancer [24], and pancreatic ductal adenocarcinoma [10]. Polymorphisms in the *PYCARD* gene appear to be associated with slightly higher frequencies of T and G allele changes than those found in Asians and the global population of allele frequency based on the gnomAD [25]. The study identified specific *PYCARD* gene variations (high T and G allele frequencies) associated with an increased risk of periodontal and cardiovascular diseases in the Iraqi population. The HWE analysis of *PYCARD* SNPs revealed no significant deviation, confirming the reliability of the genotype and allele frequencies across each study group (*P* > 0.05). In this study, periodontal indices were greater in the periodontitis groups than in the control and AS-C groups. This finding aligns with research indicating that plaque biofilms, producing a variety of bacterial by-products, trigger inflammation and accelerate the degradation of periodontal tissues [14,17,26,27,28]. In the current study, *PYCARD* gene polymorphisms were positively associated with periodontal parameters with and without atheromatous CHD. This association may indicate the presence of fundamental inflammation and molecular processes in the pathophysiology of both diseases. This study established a relationship between *PYCARD* gene polymorphisms and the development and severity of disease, suggesting that further investigation into these links could enhance our understanding of various diseases. The exclusion of prevalent risk factors for both periodontitis and cardiovascular disease, such as obesity, diabetes, smoking, and hypertension, was done in order to avert bias. To our knowledge, this is the first study to explore the association of a *PYCARD* genetic variation with susceptibility to periodontal disease, regardless of the presence or absence of coronary heart disease.

## CONCLUSION

Alterations in the T and G alleles within the TT, CT, and AG genotypes of the *PYCARD* gene were significantly associated with periodontitis and coronary heart disease, revealing complex genetic variations in the Iraqi population. The findings suggest that these specific *PYCARD* variations might influence susceptibility to both diseases, potentially through shared underlying pathways.

## Data Availability

Every genomic sequence mentioned is available at the National Center for Biotechnology Information (NCBI) Gene Bank. The specific Gene Bank accession numbers provided are LC741257, LC741259, LC741261, LC741263, LC741265, LC741267, LC741269, LC741271, LC741354, LC741356, LC741358, LC741360, and LC741361. Data supporting the conclusions of this study can be made available upon request from the corresponding author.
